# Supplementary material for: High entropy oxides for reversible energy storage
Source: Nat Commun. 2018 Aug 24;9:3400. doi: 10.1038/s41467-018-05774-5 (PMC6109100; doi:10.1038/s41467-018-05774-5)
Supplement: Supplementary file 1 — Supplementary Information [file 41467_2018_5774_MOESM1_ESM.pdf]

Supplementary Information
High entropy oxides for reversible energy storage
A. Sarkar et al

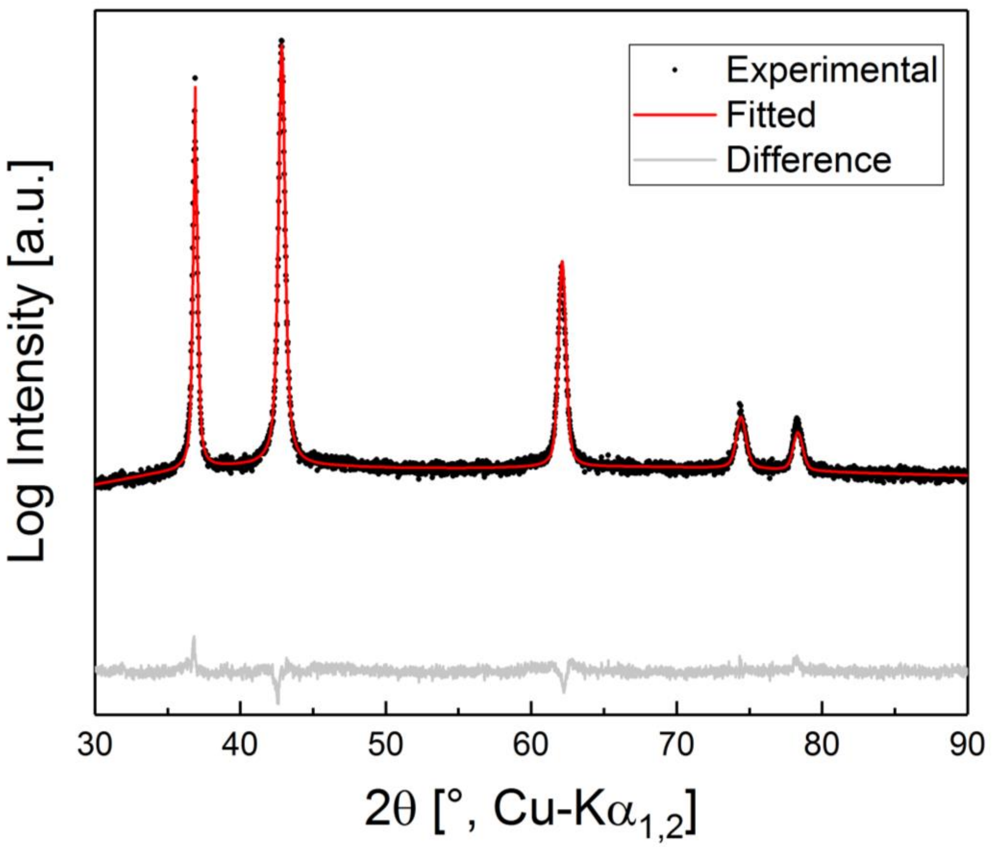

Supplementary Figure 1: Powder XRD pattern together with the corresponding Rietveld fit for TM-HEO. The goodness of fit and R_{wp} values are 1.06 and 1.39, respectively. Single phase rock-salt structure with lattice parameter of 4.2330(6) Å and average crystallite size of 36(5) nm is observed.

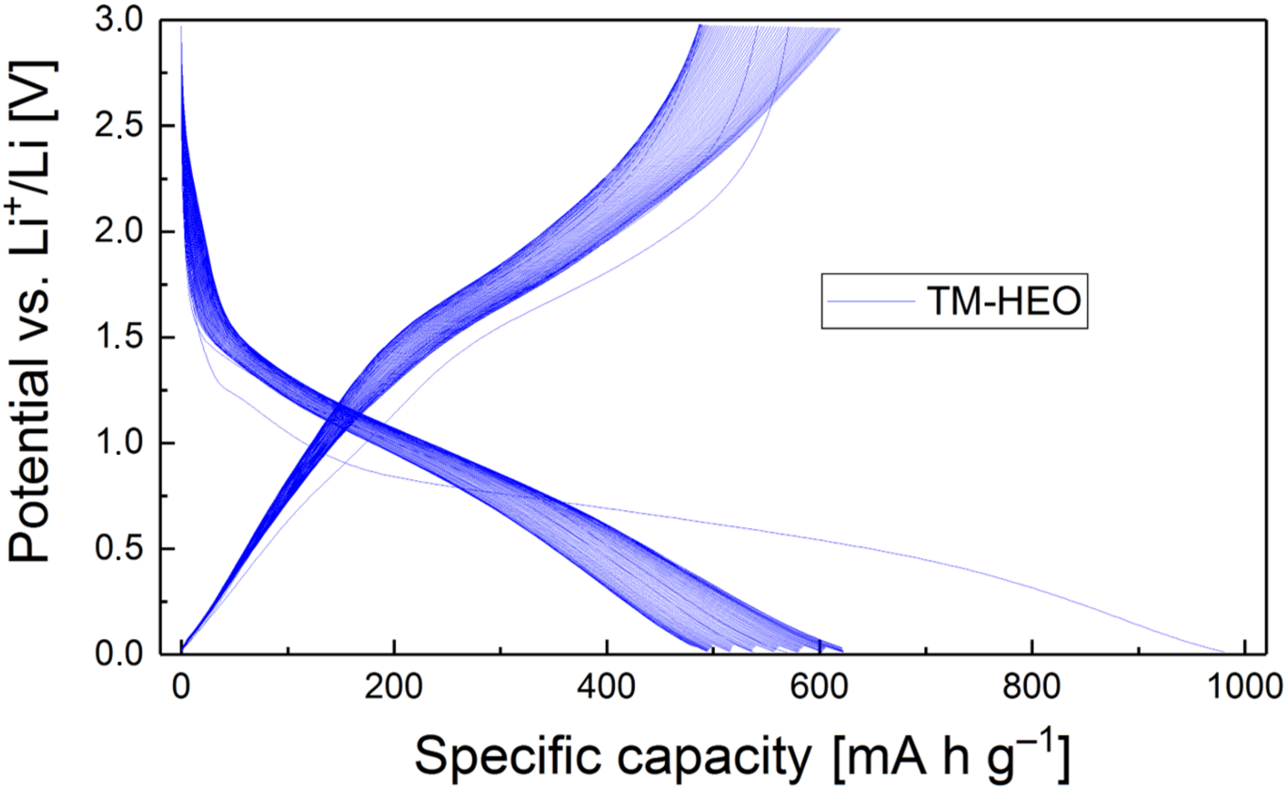

Supplementary Figure 2: First 70 voltage profiles of TM-HEO electrode operated at a current density of 200 mA/g between 0.01 and 3.0 V.

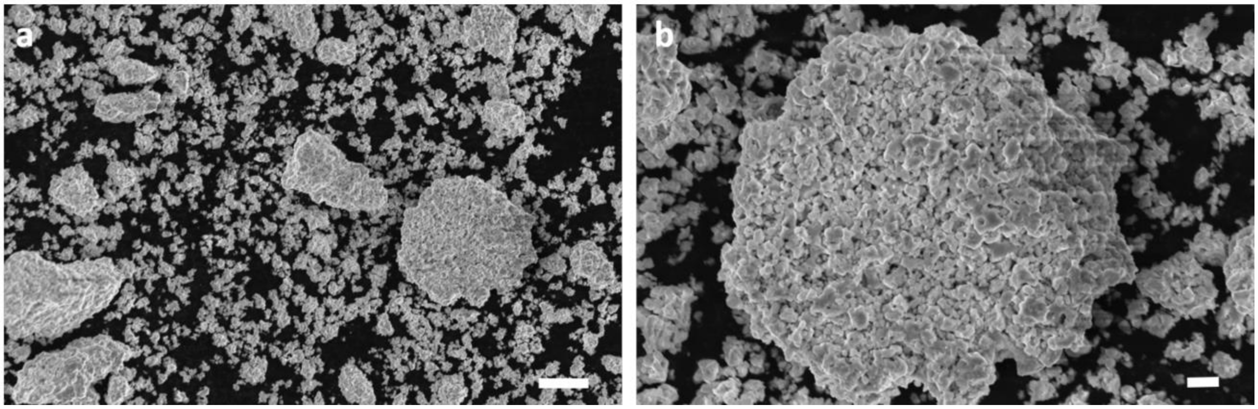

Supplementary Figure 3: SEM micrographs of sintered TM-HEO. The particles are agglomerated and larger in size. Many of the agglomerates even exceed several tens of micrometers. The scalebars are corresponding to 10 µm and 2 µm for a) and b) respectively.

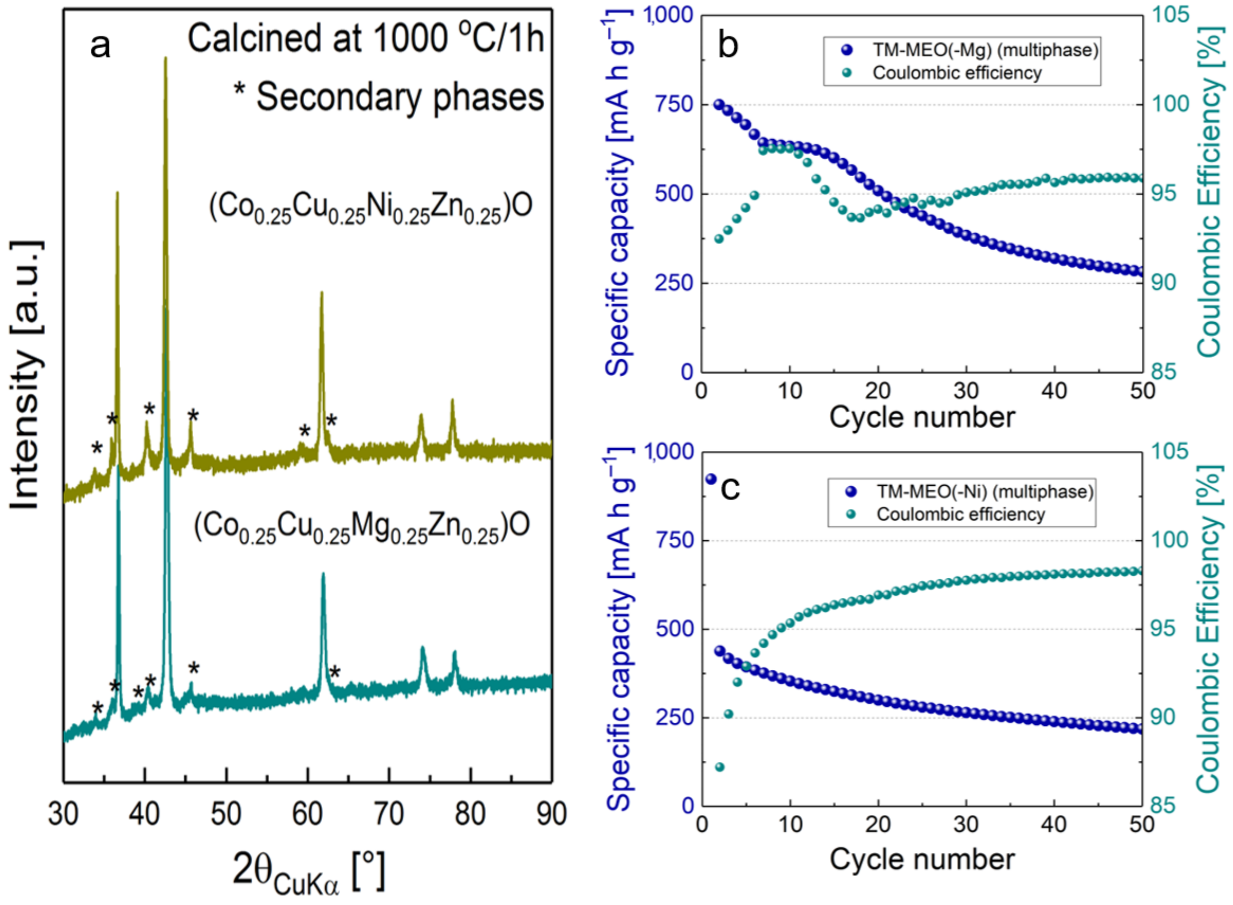

Supplementary Figure 4: Structural and electrochemical comparison of the multiphase compounds. In a) XRD patterns of multiphase compounds TM-MEO(-Ni) and TM-MEO(-Mg) are shown. These materials could not be synthesized as single phase compounds. The corresponding electrochemical characterization is shown in panels b) and c), respectively.

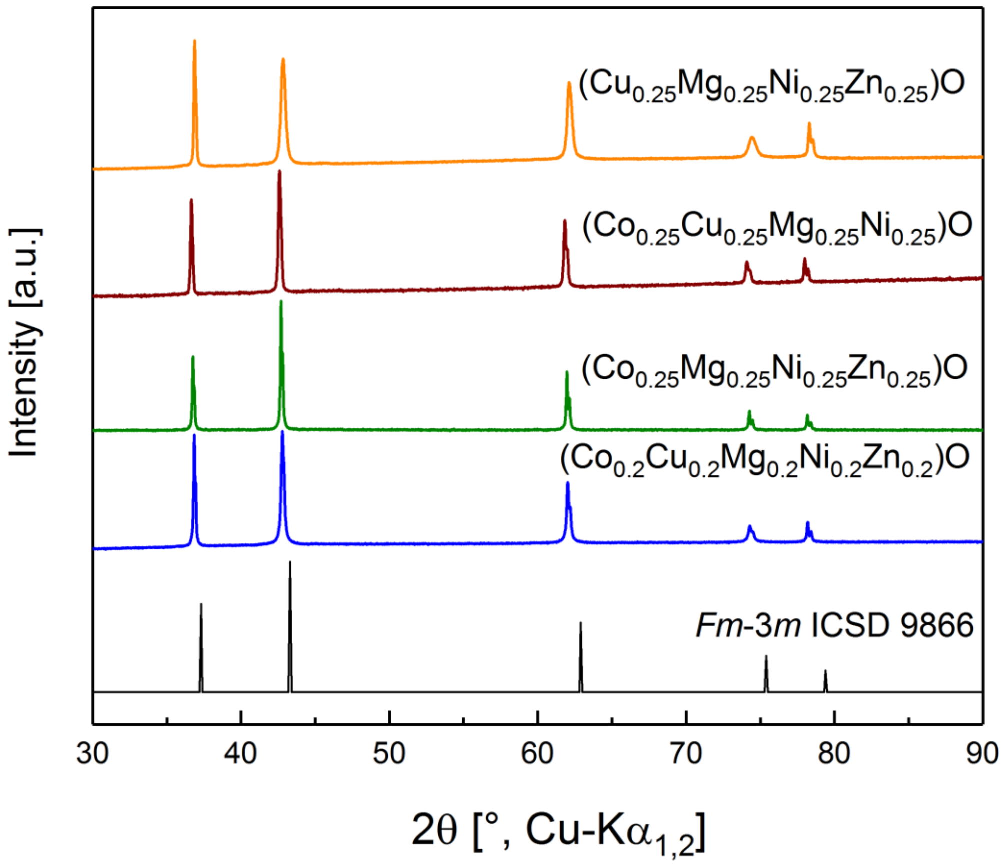

Supplementary Figure 5: Powder XRD pattern of the different materials synthesized. All of them crystallize into a single phase rock-salt structure.

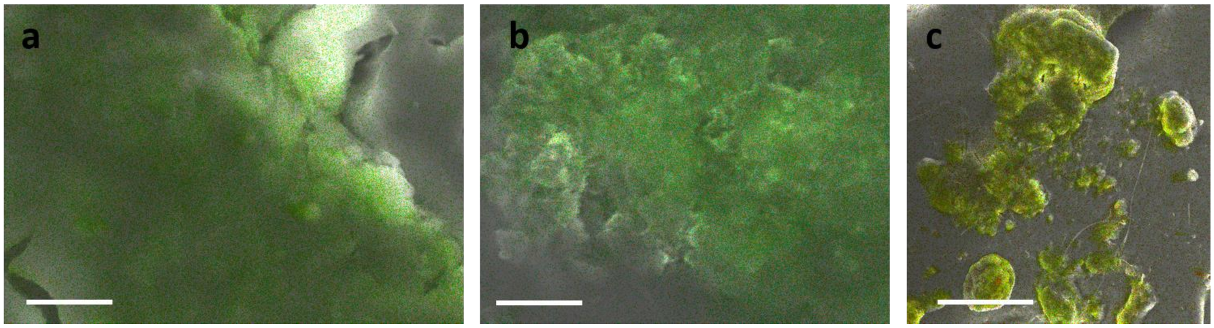

Supplementary Figure 6: EDX measurements performed on an electrode after 100 cycles. No Cu aggregation nor dendrite formation are observed. The green layer in a) and b) shows the Cu distribution. The homogenous coloring indicates that no dendrites have formed, which would appear as a higher local color intensity. In c) besides Cu (green), Ni (yellow) has been added to show the equal distribution of the different elements. The scalebars correspond to 25 µm, 10 µm and 250 µm in a, b and c respectively.

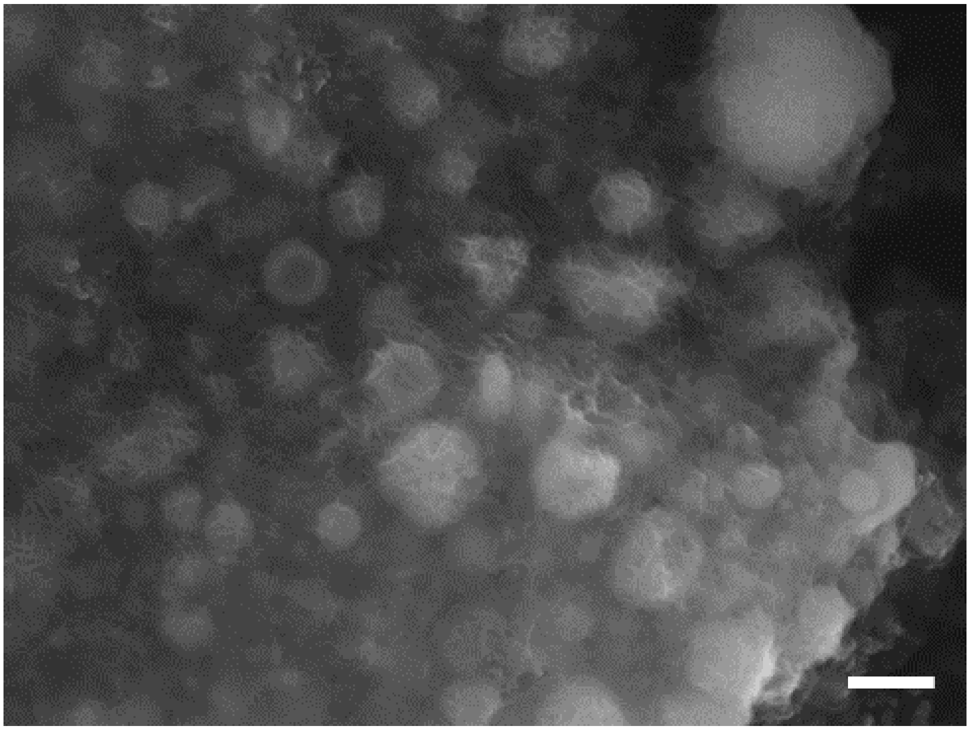

Supplementary Figure 7: SEM micrograph of the TM-HEO after several cycles, confirming that the overall morphology is maintained during cycling. This is in contrast to the typical pulverization observed for conversion-type electrodes. Scanning electron microscopy (SEM) was performed using a ZEISS Gemini Leo 1530. The scalebar corresponds to 1 µm.

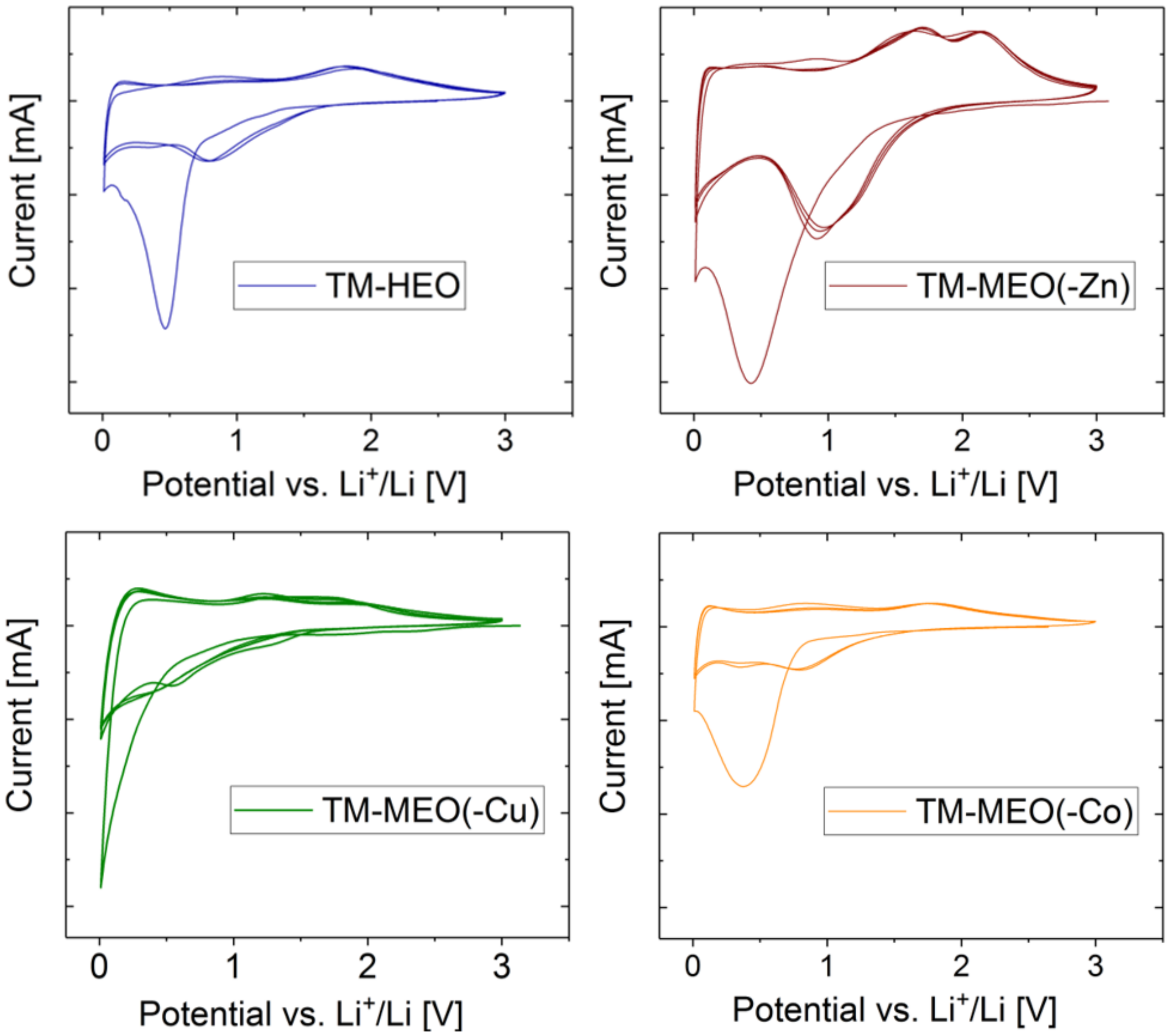

Supplementary Figure 8: Cyclic voltammograms obtained on the different single phase compounds. The curves reveal the difference in electrochemical behavior of the HEO compared to MEO. For TM-MEO(-Zn), the two anodic peaks centered around 1.7 V and 2.2 V indicate formation of NiO and CoO, respectively[2]. In the case of TM-MEO(-Cu), a distinct reduction peak at low potential appears[3].

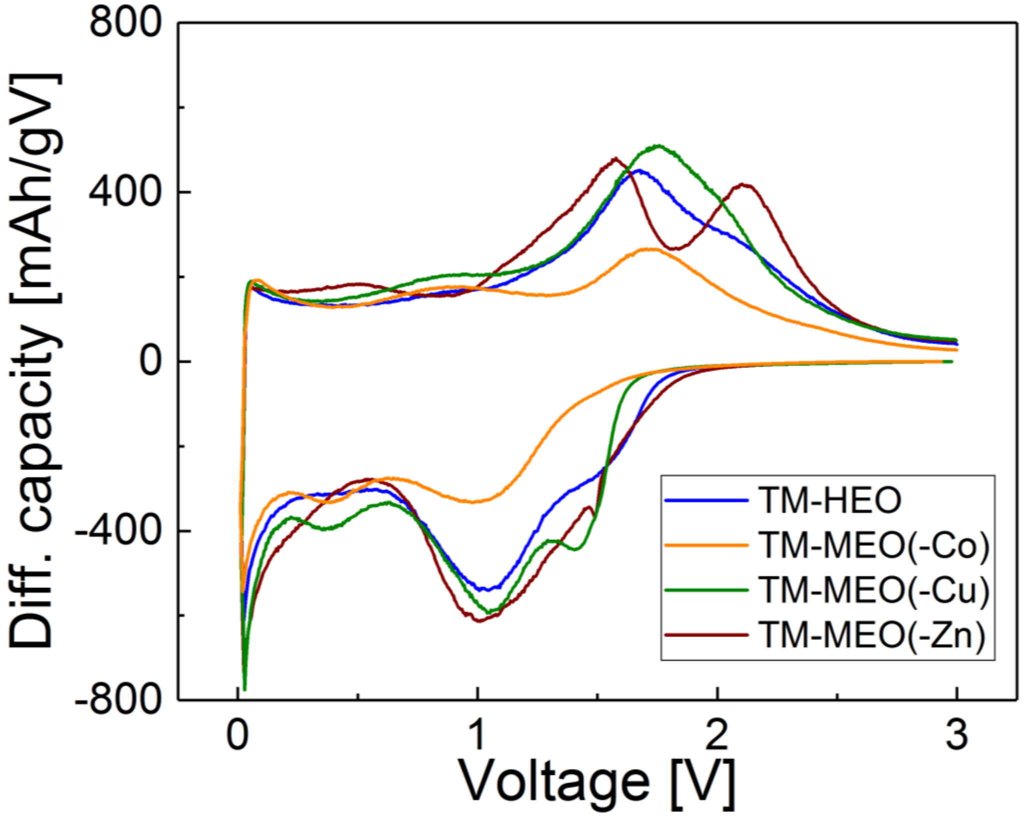

Supplementary Figure 9: Differential capacity plots for TM-HEO and the different TM-MEO compounds. It is evident that every single element has its own specific effect on the redox processes.

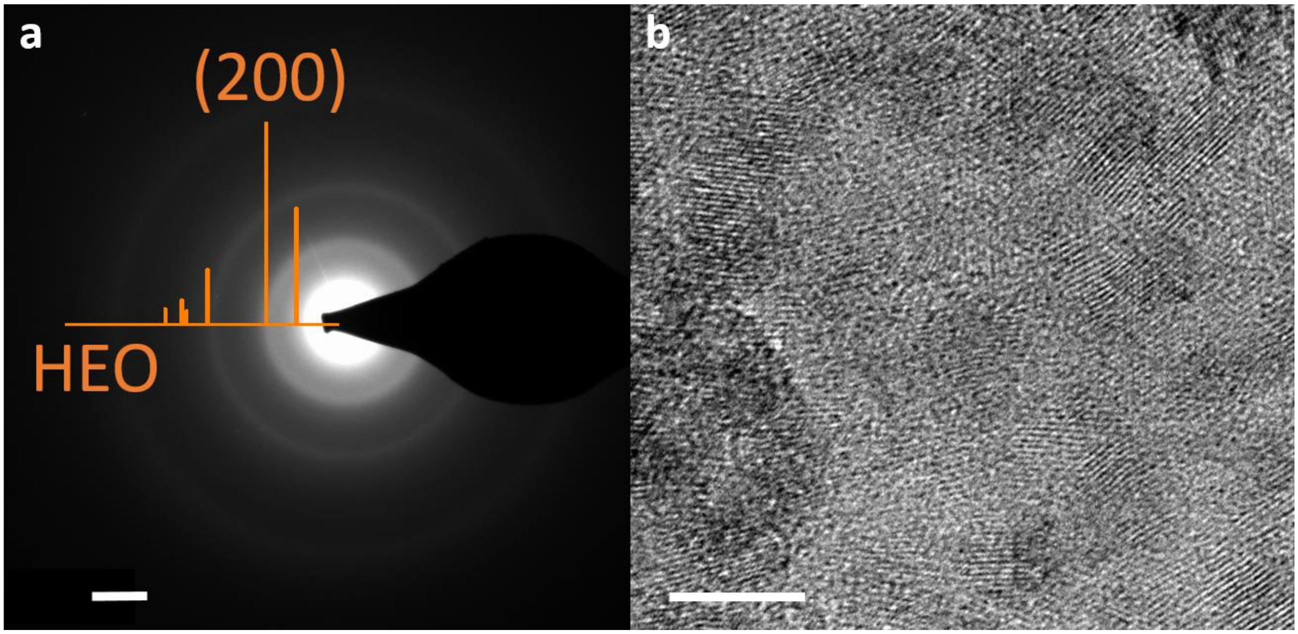

Supplementary Figure 10: TEM characterization. The SAED pattern of an electrode after 10 cycles is shown in (a), where the rock-salt structure is clearly observable. As shown, the rock-salt structure is retained, even after prolonged cycling. The presence of crystallites is clearly evident in the HR-TEM micrograph in (b). The scalebar in a) corresponds to 2 1/nm and in (b) to 5 nm.

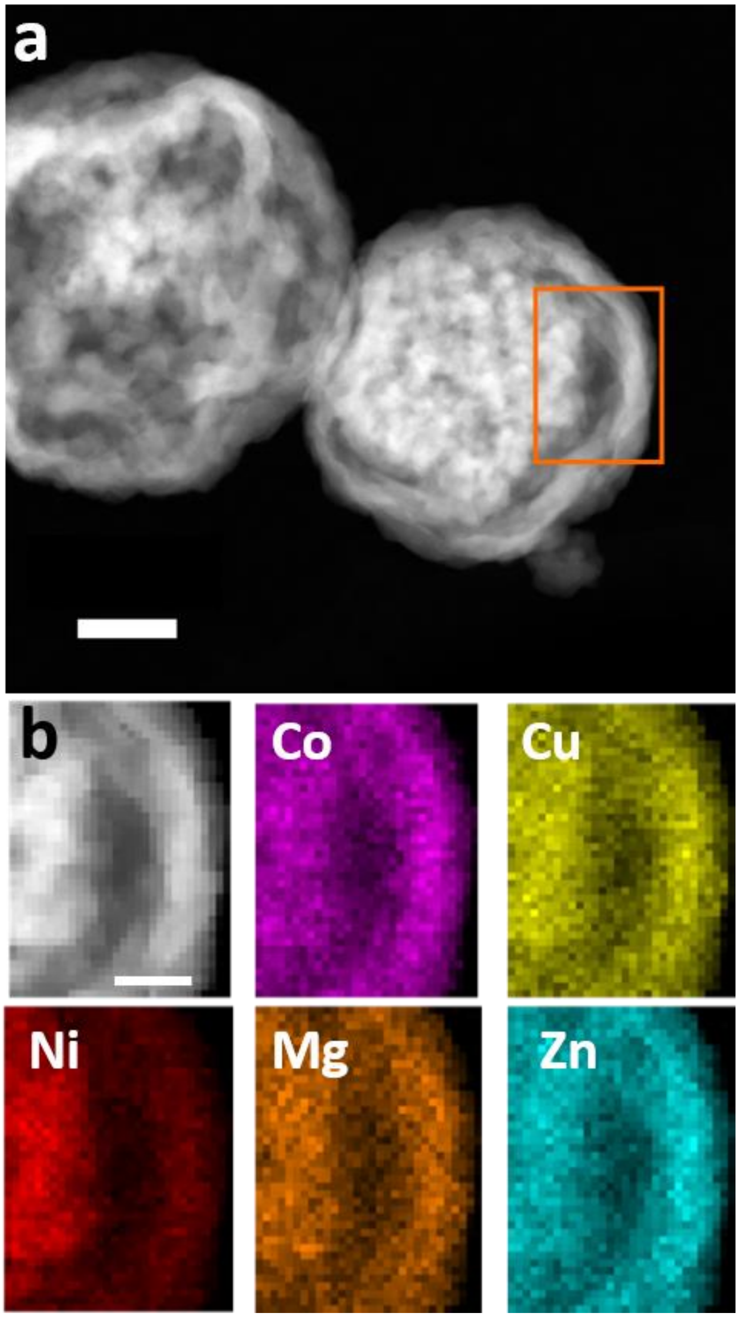

Supplementary Figure 11: STEM-EDX imaging of the as-prepared sample (orange rectangle). The scalebars in (a) and (b) correspond to 100 nm and 50 nm respectively.

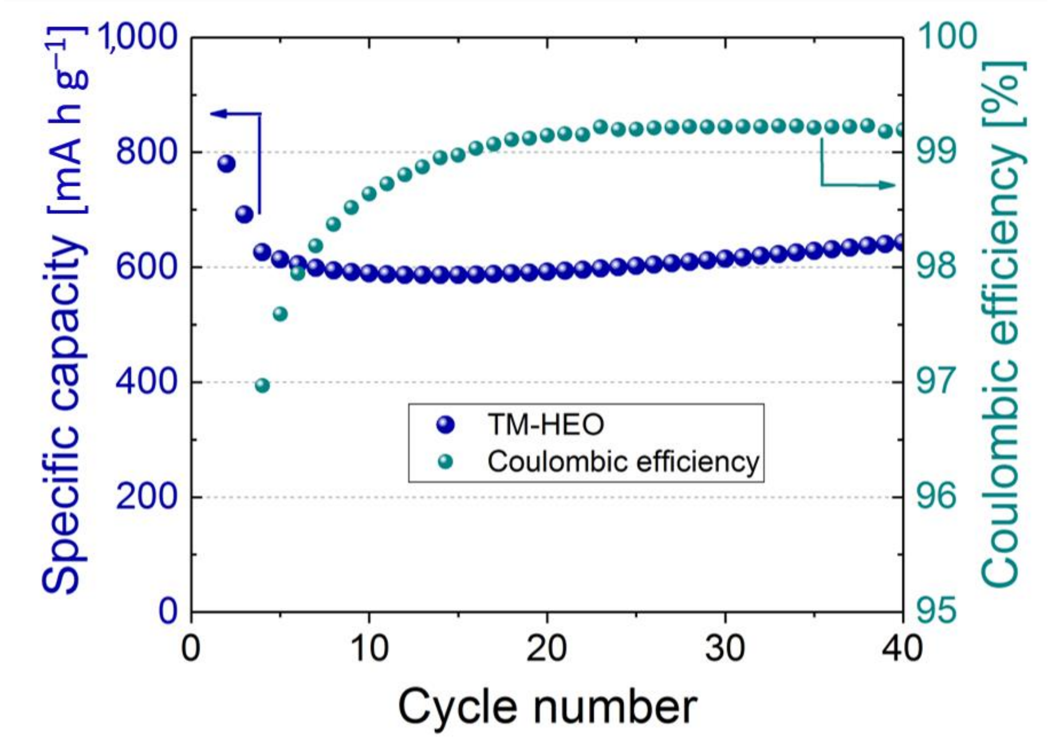

Supplementary Figure 12: Capacity retention and Coulombic efficiency of an electrode with a relatively high TM-HEO content (TM-HEO:binder:carbon black ratio of 80:10:10). The current density applied was 200 mA g^{-1}.

Supplementary Notes

The different intensity in the SAED rings displayed in Figure 3, come from a slightly different thickness of crystallites in the SAED area. The main intention to show these SAED rings is, to support the finding that a rock-salt structure is being preserved through the whole cycling process. Therefore Supplementary Figure 11a shows an SAED pattern of a 10 times cycled electrode, where it is clearly observable that the rock-salt structure can be found even after such a prolonged cycling. The nanometer-sizedcrystallites could be clearly identified in HR-TEM micrographs as can be seen in b.

Supplementary Equation 1 was used for the calculation of the theoretical capacity:

$$Q_{\mathrm{m}} = \frac{z*F}{M} \tag{1}$$

Q_{m} represents the theoretical capacity, z the number of transferred electrons, F the Faraday constant, and M is the molar mass of the active material. The specific capacity for compounds of almost the same molecular weight and redox state is very similar (e.g. $Q_{\mathrm{m}}(\mathrm{CoO}) \approx Q_{\mathrm{m}}(\mathrm{NiO}) \approx$ 715 mAh g^{-1}).

Supplementary Equation 2 was used for the calculation of the configurational entropy:

$$S_{\mathrm{config}} = -R[(\textstyle\sum_{\mathrm{i=1}}^{\mathrm{N}} x_{\mathrm{i}} \ln x_{i})_{\mathrm{cation-site}} + (\textstyle\sum_{\mathrm{j=1}}^{\mathrm{N}} x_{\mathrm{j}} \ln x_{\mathrm{j}})_{\mathrm{anion-site}}] \tag{2}$$

Example for TM-HEO with equimolar amount of different cations (5 cations → $x_{\mathrm{j}} = 0.2$):

$S_{\mathrm{config}} = -\mathrm{R}(((0.2 \ln 0.2) + (0.2 \ln 0.2) + (0.2 \ln 0.2) + (0.2 \ln 0.2) + (0.2 \ln 0.2))_{\mathrm{cation\text{-}site}} + (1 \ln 1)_{\mathrm{anion-site}})$

$S_{\mathrm{config}} = -\mathrm{R}(5 * (0.2 \ln 0.2)) = 1.61\mathrm{R}$

For the TM-MEO

$S_{\mathrm{config}} = -\mathrm{R}(4 * (0.25 \ln 0.25)) = 1.39\ \mathrm{R}$

Supplementary Method

Room temperature powder X-ray diffraction patterns were recorded using a Bruker D8 Advance diffractometer with a Cu-Kα radiation source and a LYNEXE detector having a fixed divergence slit (0.3°). Rietveld analysis (TOPAS 5, Bruker[1]) of the XRD pattern was performed to determine the crystal structure and phase composition of the as-synthesized and thermally treated powders using the structural file of NiO (Fm-3m, ICSD 9866), which was modified according to the number and type of elements present in the refined system. The instrumental intensity distribution for the XRD data was determined using LaB_6 (NIST 660a) as a reference material. Thermal displacement parameters were constrained to be equal for all the atoms.

Supplementary References

1. *Topas V5, General profile and structure analysis software for powder diffraction data, User's Manual*. (Bruker AXS, 2015).

2. Wang, Y. F. & Zhang, L. J. Simple synthesis of CoO-NiO-C anode materials for lithium-ion batteries and investigation on its electrochemical performance. *J. Power Sources* **209,** 20–29 (2012).

3. Mueller, F. *et al.* Iron-Doped ZnO for Lithium-Ion Anodes: Impact of the Dopant Ratio and Carbon Coating Content. *J. Electrochem. Soc.* **164,** A6123–A6130 (2017).
